# MALDI-TOF Mass Spectrometry Enables a Comprehensive and Fast Analysis of Dynamics and Qualities of Stress Responses of *Lactobacillus paracasei* subsp. *paracasei* F19

**DOI:** 10.1371/journal.pone.0165504

**Published:** 2016-10-26

**Authors:** Ann-Sophie Schott, Jürgen Behr, Jennifer Quinn, Rudi F. Vogel

**Affiliations:** 1 Lehrstuhl für Technische Mikrobiologie, Technische Universität München, Freising, Germany; 2 Bavarian Center for Biomolecular Mass Spectrometry, Technische Universität München, Freising, Germany; Agricultural University of Athens, GREECE

## Abstract

Lactic acid bacteria (LAB) are widely used as starter cultures in the manufacture of foods. Upon preparation, these cultures undergo various stresses resulting in losses of survival and fitness. In order to find conditions for the subsequent identification of proteomic biomarkers and their exploitation for preconditioning of strains, we subjected *Lactobacillus* (*Lb*.) *paracasei* subsp. *paracasei* TMW 1.1434 (F19) to different stress qualities (osmotic stress, oxidative stress, temperature stress, pH stress and starvation stress). We analysed the dynamics of its stress responses based on the expression of stress proteins using MALDI-TOF mass spectrometry (MS), which has so far been used for species identification. Exploiting the methodology of accumulating protein expression profiles by MALDI-TOF MS followed by the statistical evaluation with cluster analysis and discriminant analysis of principle components (DAPC), it was possible to monitor the expression of low molecular weight stress proteins, identify a specific time point when the expression of stress proteins reached its maximum, and statistically differentiate types of adaptive responses into groups. Above the specific result for F19 and its stress response, these results demonstrate the discriminatory power of MALDI-TOF MS to characterize even dynamics of stress responses of bacteria and enable a knowledge-based focus on the laborious identification of biomarkers and stress proteins. To our knowledge, the implementation of MALDI-TOF MS protein profiling for the fast and comprehensive analysis of various stress responses is new to the field of bacterial stress responses. Consequently, we generally propose MALDI-TOF MS as an easy and quick method to characterize responses of microbes to different environmental conditions, to focus efforts of more elaborate approaches on time points and dynamics of stress responses.

## Introduction

Lactic acid bacteria (LAB) play a major role in bioprocessing and are highly demanded as starter cultures in food fermentation as they are applied in many commercial products including cheeses, sourdough, sausages or wine [1–4]. In the current industrial standard, commercial concentrated starter cultures are applied as freeze dried pellets and thereby have to be reliable in terms of quality, performance and viability [5–7].

During the process of starter culture preparation and food fermentation, LAB are exposed to various unfavourable environmental conditions which affect their viability and performance. During food fermentation, LAB are confronted with several different environmental stresses like extreme values of pH, temperature, aridity and osmotic stress which affect the survival of LAB negatively by disturbing cellular viability [8, 9]. Moreover, it is known that the survival of some bacterial strains decreases during the freezing process [10, 11]. There have been approaches focusing on alternative drying processes for starter culture preparation resulting in a positively affected survival rate [12–15]. Bauer *et al*. reported that *Lactobacillus (Lb*.*) paracasei* subsp. *paracasei* TMW 1.1434 (F19) exhibits higher stability and survival rates if the strain is subjected to low-temperature vacuum drying rather than freeze drying [16].

Microorganisms, in general, are equipped with a widely spread regulatory network of stress response mechanisms to maintain cellular viability [17]. Van de Guchte *et al*. reported specifically about stress responses in LAB [7]. A common way of stress protection in LAB is the development of adaptive responses to an induced stress which, to some extent, are reflected in the proteome of bacteria. Besides, adaptive responses often induce cross-protections leading to a significant increased stress tolerance level and survival rate [18–22].

There are different methodological approaches to studying the effects of stress on LAB. As the development of adaptive responses is reflected in the proteome of bacteria, this work focuses on a proteomic approach to identify the basis of stress protection. To date, several reports describe the response of LAB to environmental changes as the expression of stress proteins which are considered to be general or specific, depending on the species, on the strains and on the type of induced stress. So, the identification of stress proteins that are related to the fitness and survival of the microorganism has so far been done by high-resolution two-dimensional (2D) gel electrophoresis (GE) coupled to Edman sequencing, MS analysis and improved computational possibilities of protein identification [23, 24]. Nevertheless, the approach of 2D GE is method-restricted since the identification and visualization of low-abundance proteins are hard to realize. Moreover, the method of 2D GE is highly demanding and time-consuming and therefore not a practical tool for screenings. The quick identification of dynamics-accompanied responses to differently induced impulses requires an easily performed tool for high sample throughput.

The widely reported method of Matrix Assisted Laser Desorption/Ionization-Time of Flight Mass Spectrometry (MALDI-TOF MS) has proven to be a powerful and reliable method to identify microorganisms. It produces spectral fingerprints with a characteristic protein expression profile of the organism analysed [25–27] and enables to investigate low molecular weight (lmw) proteins (typically 2 kDa to 20 kDa). There are studies describing the identification of small proteins of LAB to adaptive responses by MALDI-TOF MS coupled with a previous 2D GE [28, 29]. Moreover in clinical diagnostics, MALDI-TOF MS is used for protein profiling which enables the identification of biomarker molecules in tissues in order to diagnose specific illnesses [30–32]. In this study we designed an experimental setup to evaluate the methodology of MALDI-TOF MS for the fast analysis of stress responses, followed by the identification of process conditions potentially maximizing survival and fitness of a starter culture strain.

## Materials and Methods

### Bacterial strain and growth conditions

Commercial, facultatively heterofermentative *Lb*. *paracasei* subsp. *paracasei* TMW 1.1434 (isogenic with strain F19) from C. Hansen A/S (Hørsholm, Denmark) was used as a stereotype. Cells were supplied as frozen concentrate with a titer of 10^8^ cfu/ml (colony forming units) and stored at– 80°C in stocks with a final glycerol concentration of 45% (Gerbu, Heidelberg, Germany). F19 was grown anaerobically at 37°C in modified MRS (Spicher broth) [33] at pH 5.4. Standardized inocula were prepared while growing cultures overnight in fermentation broth to stationary phase, harvesting cells by centrifugation (12 000 g; 5 min; 37°C) and washing twice with phosphate buffer (0.1 M K_2_HPO_4_/KH_2_PO_4_ with 0.15 M NaCl; pH 7.0). Standardized fermentation process with an initial cell concentration of 10^7^ cfu/ml was adjusted by adding an appropriate amount of inoculum to Spicher broth. Growth was monitored by measuring the optical density at 600 nm (OD_600_) (control condition).

### Determination of sublethal stress conditions

Cultures of standardized inoculum were harvested by centrifugation (12 000 g; 5 min; 37°C) and washed twice with phosphate buffer (0.1 M K_2_HPO_4_/KH_2_PO_4_ with 0.15 M NaCl; pH 7.0). To determine growth under the influence of environmental stress, Spicher broth was modified according to different stress qualities (stress broth) (pH stress, osmotic stress, oxidative stress, starvation stress) (Table 1). Stress broth was inoculated with initial cell concentration of 10^7^ cfu/ml and incubated in final volumes of 200 μl at 37°C. Growth was monitored by measuring optical density at 600 nm in an automated microtiter plate reader (Sunrise, Tecan, Austria). The influence of temperature on growth was performed in Spicher broth at a final volume of 10 ml, incubation was performed at desired temperatures and optical density was measured at 600 nm with a spectrophotometer (Novaspec II, Amersham Biosciences, Freiburg, Germany). Samples were diluted appropriately with Spicher broth at higher optical densities.

**Table 1:** Stress qualities (SQ), stress treatments (ST) of various stress intensities (SI) subjected to *Lb*. *paracasei* subsp. *paracasei* F19 and identified sublethal stress condition (SSC).

SQ	ST	SI	SSC
**Osmotic stress**	Sodium chloride (NaCl)	0–2 M	1 M
**Osmotic stress**	Potassium chloride (KCl)	0–2 M	1 M
**Osmotic stress**	Lactose (lac)	0–0.6 M	0.32 M
**Osmotic stress**	Sucrose (suc)	0–5.8 M	1.15 M
**Oxidative stress**	Hydrogen peroxide (H_2_O_2_)	0–2 mM	1.4 mM
**pH stress**	Acid	pH 3–7	pH 4
**pH stress**	Alkaline	pH 7–10	pH 9
**Starvation stress**	Lack of glucose (glu10)	0–7.6 mM	7.6 mM
**Temperature stress**	Heat	5°C-60°C	45°C
**Temperature stress**	Cold	5°C-60°C	15°C

Further, the effect of environmental stress on growth was evaluated by determining the maximum specific growth rate μ based on the application of an induced stress (μ_stress_) and compared with experiments in control conditions (μ_max_) [34–36]. Growth rates μ were obtained by using ‘grofit’ package for RStudio software [37, 38] and were plotted against the concentration. Half maximal effective concentration (EC50) value was estimated and sublethal stress conditions were identified by choosing inhibitory concentration (IC) closest to EC50 (less than or equal in response). IC was cross-checked with the definition of sublethal stress conditions by Sanders *et al*. who described them as the reduction of the growth rate μ_stress_ to one tenth of the maximal growth rate μ_max_ [39].

### Application of sublethal stress conditions

Standardized inoculum and standardized fermentation process were carried out as described in a final volume of 10 ml. In the standardized fermentation process cells of F19 were grown to mid exponential phase, harvested by centrifugation (12 000 g; 5 min; 37°C), washed twice in phosphate buffer and subjected to sublethal stress conditions.

A sample was taken before subjecting cells to sublethal stress conditions (control). Stress samples were taken while inducing sublethal stress. The identification of dynamics of stress response based on the expression lmw stress proteins (< 20 kDa) was performed with MALDI-TOF MS and subsequently compared with the results from using 2D GE.

### Sample preparation and MALDI-TOF MS analysis

All samples were harvested by centrifugation (12 470 g; 5 min), supernatant was disposed and cells were inactivated by resuspending in ethanol (EtOH, 70%). Proteins were extracted according to the plain cell extraction protocol of Kern *et al*. [40]. Therefore, cells were harvested again by centrifugation (12 470 g; 2 min), supernatant removed, protein extracted by the application of formic acid (FA, 70%) and acetonitrile (ACN) (50:50, v/v). 1 μl suspension was transferred onto a stainless steel target and overlaid with matrix solution (10 mg/ml alpha-cyano-4-hydroxy-cinnamic acid in ACN, dH_2_O and trifluoroacetic acid (TFA) 50:47.5:2.5, v/v). All experiments were performed on different days in independent biological replicates, measured in technical triplicates. Mass spectra were obtained by a Microflex LT MALDI-TOF mass spectrometer (Bruker Daltonics, Bremen, Germany) which was equipped with a nitrogen laser (lambda = 337 nm) operating with a linear positive ion detection mode under Biotyper Automation Control 2.0 (Bruker Daltonics, Bremen, Germany). Mass spectra of each sample (mass range 2 000–20 000 Da) consisted of 400 accumulated laser shots.

### Data processing and statistical analysis

Mass spectra of each sample were exported using FlexAnalysis 3.3 (Bruker Daltonics, Bremen, Germany) and identification of stress-induced peaks was accomplished according to Usbeck *et al*. [41]. Based on an open sharedroot computer cluster (ATIX; http://opensharedroot.org) using a self-tailored MASCAP [42], which was implemented in octave software [43–45], all exported mass spectra of each sample were pre-processed by subtracting the baseline, smoothing and normalizing signal intensities. The pre-processed mass spectra were used for peak detection by picking peaks which show the highest intensity among their nearest points. Alignment and subsequent clustering of stress-induced peaks were performed with 600 ppm as limit of distance tolerance [41, 42].

Peak-based cluster analysis was carried out to visualize dynamics of stress responses, and especially, to determine a specific time point when the expression of lmw stress proteins reached its maximum. Therefore, hierarchical cluster analysis was performed by using the ‘hclust’ package for RStudio software. Based on the calculation of the distance matrix, using the method ‘manhattan_bc’, hierarchical clustering was carried out with the method ‘complete linkage’ and visualized via dendrogram.

Furthermore, differentiation of stress responses was accomplished by a mathematical approach based on discriminant analysis of principal components (DAPC) using the ‘adegenet’ package for RStudio software [46]. The approach of DAPC was described in detail in [41] and is summarized in the following. DAPC checked for optimal grouping of evaluated MALDI-TOF mass spectra by optimizing the variance between groups while neglecting within-group-variation. Therefore, optimal clusters were identified using ‘find.clusters’ and subsequently ‘dapc’ was executed based on groups defined previously by ‘find.clusters’. Additional grouping of stress responses was accomplished by alternative labelling. In consequence, DAPC finally provides a barplot of eigenvalues and a scatterplot representing individuals as dots and groups as inertia ellipses.

### 2D gel electrophoresis and analysis

In order to confirm the approach of using MALDI-TOF MS protein profiling for the analysis of stress responses and thus the identification of process conditions maximizing survival and fitness of a starter culture strain, protein patterns were additionally examined with 2D GE. Since this method is highly time-consuming, we focused on the comparison of protein patterns of control and of a defined, sublethal stress condition that was previously detected by MALDI-TOF MS coupled to cluster analysis.

Standardized inoculum and standardized fermentation process were carried out as described in a final volume of 10 ml. In the standardized fermentation process cells of F19 were grown to mid exponential phase, harvested by centrifugation (12 000 g; 5 min; 37°C), washed twice in phosphate buffer and subjected to a defined, sublethal stress condition. Control sample was taken before subjecting cells to defined, sublethal potassium chloride stress (1 M KCl, 60 min). Whole cell proteins of control and stress sample were extracted according to Görg *et al*. with minor modifications [47]. The cell walls were digested with 500 kU lysozyme in lysozyme buffer (5 mg/ml lysozyme, 0.5 M sucrose in TE buffer) for 60 min at 37°C. Digested cells were centrifuged, supernatant discarded, pellet washed twice in ultrapure water and finally resuspended in 200 μl SDS buffer (0.9% SDS, 0.1% Pefabloc, 100 mM Trisbase, pH 8.6). The cell disruption was performed by sonication (HD—70/Bandelin; 7 cycles of 30 s, power 90%, cycle 30% and intermediate cooling). Lysed cells were diluted 2.5 fold with CHAPS lysis buffer (6.71 M urea, 1.79 M thiourea, 65.06 mM CHAPS, 1% w/v DTT, 0.5% v/v Pharmalyte 3–10) and proteins solubilized by adequately mixing for 20 min. Cellular debris was excluded by centrifugation (17 500 x g, 4°C, 30 min) and the protein lysate was stored at– 80°C.

Isoelectric focusing (IEF) was performed in a flat-bed isoelectric focusing unit (IEF100 First-dimension Isoelectric Focusing Unit, Hoefer Inc., Holliston, MA, USA) with 24-cm immobilized-pH-gradient (IPG) 4 to 7 strips (SERVA IPG BlueStrip, Serva Electrophoresis GmbH, Heidelberg, Germany) at 20°C. IPG strips were rehydrated in CHAPS rehydration buffer (6.71 M urea, 1.79 M thiourea, 8.13 mM CHAPS, 0.2% w/v DTT, 0.2% v/v Pharmalyte 3–10) and 200 μg of protein was subsequently applied by anodic cup loading. Initial IEF was gradually performed twice, each time for 6 h to 250 V. IEF to steady-state at 10 000 V was carried out according to the micro-preparative IEF protocol [47]. SDS-polyacrylamide gel electrophoresis (SDS-PAGE) was carried out on vertical gel electrophoresis unit (SE900 Large Format Vertical Gel Electrophoresis Unit, Hoefer Inc., Holliston, MA, USA) with gels of total acrylamide concentration of T12% at 15°C. The proteins were visualized by silver staining [48], protein patterns analysed and compared using Prodigy 2D (Nonlinear dynamics Limited, Newcastle, UK). The effect of an induced stress was considered if the mean normalized spot volume (MNSV) varied at least 1.4-fold and confirmed by analysis of variance at a significance level of P < 0.05. All experiments were performed on different days in independent biological triplicates, measured in technical triplicates.

### Reference genome sequencing for protein identification

A reference genome was established in order to identify protein spots derived from 2D GE. Therefore, high molecular weight DNA of F19 was isolated using the Genomic-tip 100/G (Qiagen, Venlo, Netherlands) kit according to the manufacture with minor modifications. Cell lysis was performed with exponentially grown cells and was adapted with respect to lysis time. An exceeded lysis time to maximally 16 h was sufficient for obtaining a clear lysate. Isolated DNA was sequenced at GATC Biotech (Konstanz, Germany) by SMRT sequencing [49, 50] and the complete genome of F19 was assembled, annotated and submitted to GenBank [51, 52].

### MS protein characterization

The silver stained protein spots were excised from the gel and sent to the Zentrallabor für Proteinanalytik (Ludwig-Maxmimilians-Universität München, Munich, Germany) for electrospray ionization (ESI) tandem MS (MS/MS).

### Protein identification

For protein spots identification, all MS/MS samples were analysed using Mascot (Matrix Science, London, UK; version 2.3.02) and X! Tandem (The GPM, thegpm.org; version CYCLONE (2010.12.01.1)). Mascot and X! Tandem were set up to search the strain-specific database containing coding sequence translations of the sequenced genome of F19 (2938 entries) assuming the digestion enzyme trypsin. Mascot and X! Tandem were searched with a fragment ion mass tolerance of 0.75 Da and a parent ion tolerance of 25 PPM. Carbamidomethyl of cysteine was specified as a fixed modification in Mascot and X! Tandem. Glu->pyro-Glu of the n-terminus, ammonia-loss of the n-terminus, gln->pyro-Glu of the n-terminus, oxidation of methionine and acetyl of the n-terminus were specified as variable modifications in X! Tandem. Whereas, oxidation of methionine and acetyl of the n-terminus were specified as variable modifications in Mascot.

Validation of MS/MS based peptide and protein identifications of 2D protein spots was achieved by Scaffold (version Scaffold_4.6.1, Proteome Software Inc., Portland, OR). Peptide identifications were accepted if they could be established at greater than 95.0% probability by the Scaffold Local FDR algorithm (74.3% Decoy Peptide FDR). Protein identifications were accepted if they could be established at greater than 99.0% probability and contained at least 2 identified peptides (97.1% Decoy Protein FDR). Protein probabilities were assigned by the Protein Prophet algorithm [53]. Proteins that contained similar peptides and could not be differentiated based on MS/MS analysis alone were grouped to satisfy the principles of parsimony. Proteins sharing significant peptide evidence were grouped into clusters.

## Results

### Determination of sublethal stress conditions

In order to identify sublethal stress conditions, we subjected F19 to different stress qualities (osmotic stress, oxidative stress, temperature stress, pH stress and starvation stress) and the OD of the culture was measured at 600 nm.

Measured growth in all applied stress broths exhibited a common pattern in which F19 displayed characteristic growth curves at low stress intensities, whereas it showed minimal to no increase in the optical density the higher the stress intensity applied. Compared to growth in stress broth, control experiments exhibited, with two exceptions (oxidative stress, pH stress), highest optical densities. F19 entered the exponential phase after 2 hours (h) of fermentation and reached its maximum specific growth rate μ_max_ of 0.3 in mid exponential phase. After 10 h growth in Spicher broth, cells of F19 passed into stationary phase.

**Fig 1:**
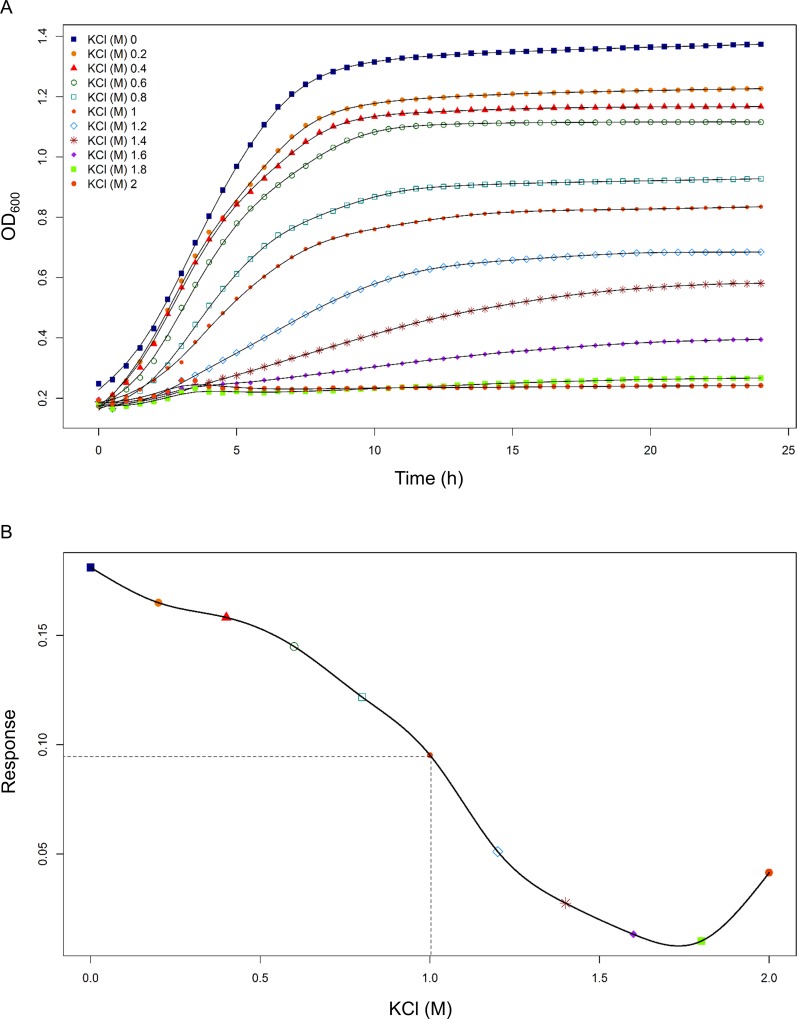
Growth curve analysis of *Lb*. *paracasei* subsp. *paracasei* F19. (A) Growth in control conditions and in stress broth of different concentrations of potassium chloride. (B) Growth rate μ_stress_ plotted against concentration (M).

As an example, the effect of potassium chloride stress on growth of F19 is depicted in Fig 1A. In stress broth enriched with 1.2 M to 2 M KCl, F19 showed no to minimal growth up to a maximal OD (OD_max_) of 0.6. In stress broth of 1 M to 0.2 M KCl, OD_max_ increased from 0.8 up to 1.2. As previously described, growth in control conditions compared to growth under potassium chloride stress was highest with OD_max_ of 1.4 in stationary phase. The specific growth rate was calculated and plotted against the concentration of potassium chloride, and EC50 for F19 was identified at 1 M KCl. According to Sanders *et al*. (μ_stress_ = 1/10 μ_max_) sublethal stress condition was identified at 1.0 M KCl (Fig 1B) along with identified IC.

For F19, sublethal stress conditions were identified for all stress treatments as described above and summarized in Table 1.

### MALDI-TOF MS analysis

MALDI-TOF mass spectra were recorded for *Lb*. *paracasei* subsp. *paracasei* F19 under control and sublethal stress conditions within a mass to charge ratio (m/z) of 2 000 to 20 000. Spectra showed only slightly visible differences in protein expression profiles. Control and stress-induced samples exhibited most differences in peak intensity along with the duration of the induced stress (S1 Fig).

**Fig 2:**
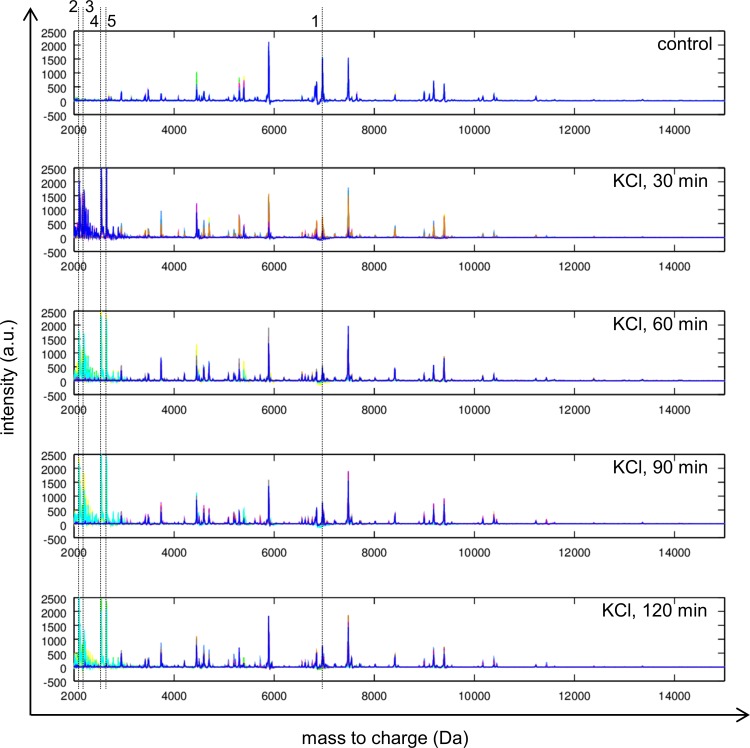
MALDI-TOF mass spectra obtained for *Lb*. *paracasei* subsp. *paracasei* F19 in the mass range from 2 000 Da to 15 000 Da. Control conditions (control) and sublethal potassium chloride at different sampling points are indicated. Dotted line 1 marks a single peak at approx. 6 991 m/z (1) with decreasing peak intensity. Dotted lines 2, 3, 4 and 5 mark peaks at 2 106 m/z (2), 2 122 m/z (3), 2 549 m/z (4) and 2 649 m/z (5) with increasing peak intensity.

Mass spectra of control and obtained under sublethal potassium chloride stress illustrating differences are depicted in Fig 2. Peaks with approx. 6 991 (1), 2 106 (2), 2 122 (3), 2 549 (4) and 2 649 (5) m/z changed in intensity with the presence of 1 M KCl. Noticeably, peak 1 decreased in intensity and visibly reached its minimum at the sampling point of 60 minutes to 500 arbitrary units (a. u.). With increasing duration of stress induction, peak 1 raised again in peak intensity, but did not reach its maximum compared to the control. Furthermore, in the presence of 1 M KCl at any sampling point, numerous peaks were detected (2, 3, 4, 5) which clearly increased in their peak intensity. Especially peak 2, peak 4 and peak 5 showed massive enhancements in peak intensity up to 2 500 a. u. while they were not visible in control conditions.

The peak-based hierarchical clustering of 477 MALDI-TOF mass spectra was performed. In order to examine differences or similarities and thus detecting changes on the proteome level, cluster analysis was done for each stress treatment taking the corresponding sublethal stress and control spectra into account. For all hierarchical cluster analyses, mass spectra of the control (standard) were grouped in a single arm (S2 Fig) showing a low level of similarity to the analysed stress samples which were harboured in a distinct cluster. Furthermore, the mass spectra of the stress samples were grouped along with the duration of the induced sublethal stress.

The cluster analyses of sublethal sodium chloride (NaCl), alkaline pH (pH9) and lactose (lac) stress are shown in S2 Fig, demonstrating analogies among the cluster formation. The analysed mass spectra of the stress samples were separated, based on the sampling points, in two sub-clusters. Concerning the stress treatments with sodium chloride or alkaline pH, one sub-cluster harboured spectra of 90 and 120 minutes duration of stress induction (late phase of stress response), demonstrating higher variability to each other and any other sampling point of the stress response. Thus, the other sub-cluster consisted of 30 and 60 minutes stress application. Regarding the stress treatment with lactose, the stress responses of 60 and 120 minutes duration of stress induction grouped in a sub-cluster revealing higher similarity and low distance to each other than to the other sub-cluster, which harboured stress responses of 30 and 90 minutes of stress induction.

The hierarchical clustering of glucose-starvation (glu10), potassium chloride (KCl), acidic pH (pH4), sucrose (suc), hydrogen peroxide (H2O2) or temperature (15°C, 45°C) stress treatment grouped the analysed mass spectra of the stress samples in solely one cluster (S2 Fig). In case of glucose-starvation, potassium chloride or acidic pH stress, the analysed mass spectra of 60 and 90 minutes duration of stress induction showed lowest distance to each other than to the rest of the sampling points—30 minutes and 120 minutes duration of stress application. For sucrose or heat stress (45°C), the stress responses of 90 and 120 minutes duration of stress induction displayed a lower distance to each other and thus indicated a higher similarity to each other than to stress responses after 30 and 60 minutes of stress induction. Regarding the stress treatment with hydrogen peroxide, the lowest distance of the cluster was detected for stress responses of 30 and 90 minutes duration of stress induction, whereas the cluster analysis of cold stress (15°C) detected the highest similarity between stress responses of 60 and 120 minutes of stress induction.

**Fig 3:**
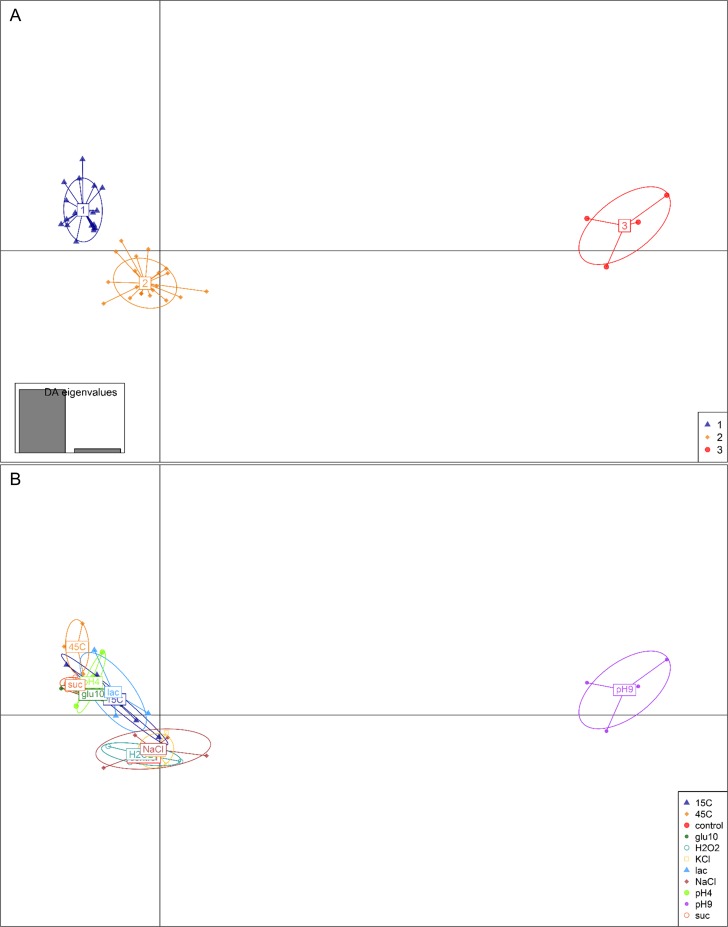
The DAPC analysis performed with the ‘adegenet’ package for the R software, displayed as a scatterplot of the first 2 principle components. 477 MALDI-TOF mass spectra were considered. (A) Optimal clusters defined by ‘find.clusters’. (B) Data labelled according to induced sublethal stress conditions.

Besides monitoring the dynamics of stress responses via cluster analysis, the differentiation of these stress responses in groups was additionally performed using Discriminant Analysis (DA) along with Principal Component Analysis (PCA). Applying ‘find.clusters’ from the R package ‘adegenet’, 3 optimal clusters were identified using the first two PCs explaining 80% of the cumulative variance. The same data were additionally stress labelled. The provided scatterplot enabled a graphical estimation of the structures between groups. In Fig 3, stress responses optimally clustered together in three distinct groups.

The comparison of the optimal clusters with the stress labelled data revealed that cluster 3 corresponds to the stress response to alkaline pH stress (pH9), cluster 2 harbours stress responses to sodium chloride (NaCl), potassium chloride (KCl), hydrogen peroxide (H2O2) and control conditions and cluster 1 covers stress responses to lactose (lac), sucrose (suc), acidic pH (pH4), heat (45°C), cold (15°C) and glucose-starvation (glu10) stress. One outlier of the grouped stress response to cold stress (cluster 1) overlapped with the ellipse of the grouped stress response to sodium chloride (cluster 2). Looking at the stress labelled data, stress responses to acidic pH and alkaline pH clearly differed from each other. Furthermore, stress response to sublethal lactose (lac) and sucrose (suc) stress lapped over while they differed from sublethal sodium (NaCl) and potassium chloride (KCl) stress.

### Differential proteome analysis

**Fig 4:**
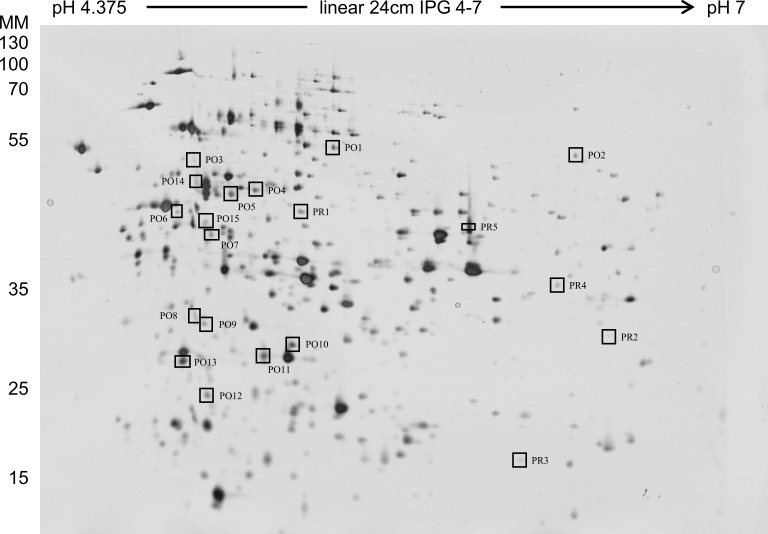
2D electrophoresis analysis of silver stained whole cell proteome of *Lb*. *paracasei* subsp. *paracasei* F19. P, grown under sublethal potassium chloride stress. O, proteins overexpressed in potassium-stressed cells. R, proteins repressed in potassium-stressed cells.

**Fig 5:**
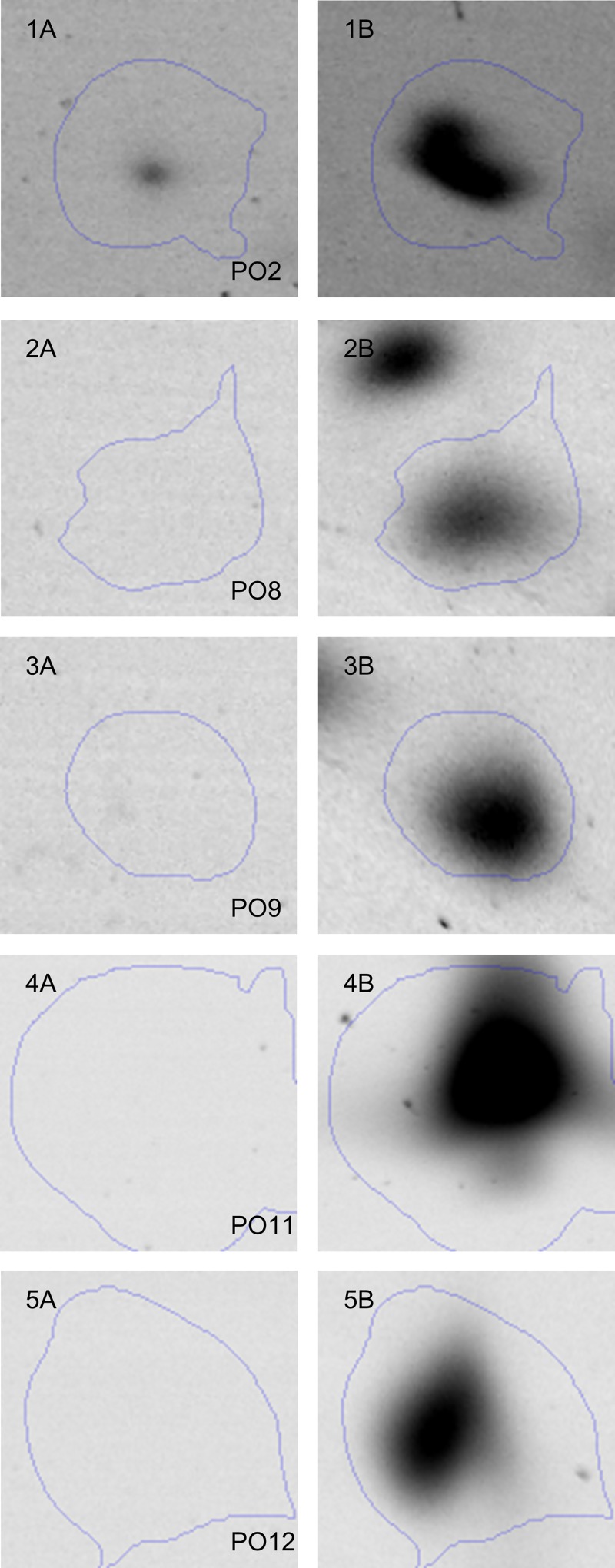
Expression profiles of protein spots. Protein spots PO2 (1), PO8 (2), PO9 (3), PO11 (4) and PO12 (5) of *Lb*. *paracasei* subsp. *paracasei* F19 in control (A) or under defined sublethal potassium chloride (1 M, 60 min) conditions (B).

Whole cell proteins of *Lb*. *paracasei* subsp. *paracasei* F19 were extracted under control conditions and under defined potassium chloride stress (1 M KCl, 60 min). High-resolution separation of the whole cell proteome was achieved using immobilized pH gradient (IPG) strips with 24 cm separation distances in the range from pH 4 to 7 in the first dimension, followed by the separation of the molecular mass in the range from 10 to 250 kDa in the second dimension. The comparison of the protein patterns revealed that the induction of defined, sublethal potassium chloride stress led to significant differences in expression levels of overall 20 protein spots (Fig 4). Among these protein spots, five spots were repressed (PR1-5), whereas 15 were overexpressed (PO1-15) in the presence of defined, sublethal potassium chloride stress. The expression profile of the strongly affected potassium chloride overexpressed protein spots PO2 (1.9-fold MNSV, p = 0.007), PO8 (1.8-fold MNSV, p = 0.001), PO9 (1.7-fold MNSV, p = 0.018), PO11 (1.9-fold MNSV, p = 0.003) and PO12 (1.7-fold MNSV, p = 0.012) are depicted in Fig 5.

### Protein identification of 2D protein spots

Protein analysis of 2D protein spots was performed by mass spectrometry. Strongly affected protein spots PO2, PO8, PO9, PO11 and PO12 were subjected to ESI MS/MS analysis. MS/MS data were searched using Mascot and X! Tandem against the strain-specific database (see above) and identified proteins were validated by Scaffold. In total, 270 proteins were accepted in accordance to the minimum peptide number, the peptide and protein threshold. Considering a high protein identification probability, the sequence coverage, the immobilized pH gradient (pH 4–7) and the molecular mass range, probable proteins are displayed in Table 2. Further information regarding peptide sequences and the total number of unique peptides are listed in S1 Table. Proteins regulated under potassium chloride stress were enzymes from the secondary metabolites biosynthesis, transport and catabolism (CTP-synthase), the amino acid metabolism (proline iminopeptidase), the amino acid metabolism and transport (glutamine ABC transporter ATP-binding), and the DNA metabolism (single-stranded DNA-binding protein). Furthermore, the osmotic shift induced alanine-phosphoribitol ligase, which could not be designated any function.

**Table 2:** Probable proteins identified from 2D gel electrophoresis. Displayed are the analysed protein spots from 2D gel electrophoresis (sample), the probable protein identifications (Protein name) including the protein identification probability (%) and sequence coverage (%) in accordance to the accession no., molecular weight (Mol wt) (kDa) and pI value of each protein.

Spot	Protein name	Accession no.	Mol wt (kDa)	pI value	Protein identification probability (%)	Sequence coverage (%)
**PO2**	CTP synthase	BBD24_12645	59.78	6.00	100.0	12.7
**PO8**	proline iminopeptidase	BBD24_12800	33.39	4.85	100.0	24.7
**PO9**	alanine-phosphoribitol ligase	BBD24_05660	34.77	5.19	100.0	11.7
**PO11**	glutamine ABC transporter ATP-binding protein	BBD24_07020	27.30	4.98	99.6	4.4
**PO12**	single-stranded DNA-binding protein	BBD24_00055	21.14	5.14	100.0	17.2

## Discussion

This study demonstrates the power of MALDI-TOF MS protein profiling to differentiate stress intensities and qualities in a fast and easy approach. Microbial responses to sublethal stress conditions were examined and changes on proteome level were detected.

So far, the proteomic approach of high-resolution 2D GE has most commonly been used in order to provide an insight into general versus specific stress response [20]. Regarding a fast screening of stress responses, the approach of 2D GE has its limitations and forces the evaluation of an easily manageable tool for high sample throughput.

MALDI-TOF was introduced in the field of mass spectrometry in 1990 [54, 55] and has allowed various forms of application since then. Its ability to perform proteome profiling has on the one hand mostly been used for rapid species identification in order to assure product quality and safety in food microbiology and biotechnology [56–59], and on the other hand to identify pathogens in clinical diagnostics [60–62]. Especially in clinical diagnostics, the implementation of MALDI-TOF MS profiling enables the identification of biomarker molecules in tissues in order to diagnose specific illnesses [30–32]. In this work we exploited the MALDI-TOF MS profiling technique as a tool for analysing microbial stress responses based on the accumulation of lmw stress proteins in order to provide a basis for the identification of biomarkers which help the microorganisms to increase their fitness and thus to survive harsh environmental conditions.

Protein expression profiles were recorded for control experiments and sublethal stress conditions. A closer look at the obtained protein expression profiles displayed differences in peak intensity and mass depending on the sublethal stress conditions induced. Global stress peaks were not detected. Nevertheless, visual changes in peak intensity at specific m/z were found within each stress treatment while inducing sublethal stress. Changes in the protein expression profiles under varying stress conditions are logical, since it is known that the induction of sublethal stress is reflected in the proteome of microorganisms [7]. Since the acquisition of MALD-TOF mass spectra is attributed to the detection of ribosomal and cell structure proteins which are resembled as peaks [63], it is additionally reasonable that varying characteristic peaks, which were connected to the induced stress treatment, are attributed to the organism’s stress response. A former study of our group confirmed these findings by detecting characteristic acid and hop shock induced responses in beer spoiling *Lactobacillus brevis* by MALDI-TOF MS coupled to ESI MS/MS [64].

Besides, we demonstrated the ability of MALDI-TOF MS protein profiling coupled to peak-based cluster analysis to display similarities and differences (within sublethal stresses) of stress response intensities over a period of time. Hierarchical clustering was performed with all protein expression profiles and stress response of F19 turned out to be distinct from control all along. Based on the expression of lmw protein, the microbial stress response along the stress duration varied for all sublethal stress conditions. Overall, stress responses, which were grouped in clusters, showed lowest distance to each other and thus supported a close relationship. Considering this, it is possible to differentiate stress responses mainly in an early (30–60 min), middle (60–90 min) and late (90–120 min) phase of stress response. In consequence, a specific time point when the expression of lmw stress proteins reaches its maximum is assumed for minimally 60 or maximally 90 minutes of stress induction depending on the applied stress treatment. In the presence of environmental stresses, microorganisms activate a regulatory network which controls cellular protein quality [65, 66]. Native proteins start to misfold and degrade. In consequence, a microbial protein control system adjusts the cellular level of chaperones and proteases which are already present to prevent protein aggregation [67]. According to that, the visually detectable changes in peak intensities could be attributed to microbial stress responses illustrating dynamic changes of stress proteins along the application of sublethal stresses. Several studies describe stress responses of microorganisms in general and reveal dynamics of stress responses after an induced environmental stress or stimuli by reacting in short- and long-term responses on transcriptome and metabolic level [68–70]. Dynamics of stress responses on proteome level are less known. Van de Guchte *et al*. reviewed that short-term stress responses primarily lead to the activation or stabilization of small proteins which are already present [7], whereas long-term adaptation processes to environmental stress evoke changes in membrane compositions [71]. Furthermore, the ability to quickly respond to environmental stress is essential for LAB in order to survive [72]. Thus, the implementation of MALDI-TOF MS protein profiling enables the detection of dynamics of stress responses and thereby the expression of lmw stress proteins along the application of sublethal stresses. Besides that, it might be interesting to use MALDI-TOF MS protein profiling as a replacement for the procedure to identify sublethal stress conditions by solely focusing on the reduction of growth rates. In consequence, MALDI-TOF MS analysis provides a powerful tool to identify maximal responses and thus enables the analysis of the state of fitness based on the proteome.

The methodology of DAPC allows the visualisation of microbial responses, here of the model organism *Lb*. *paracasei* subsp. *paracasei* F19, to various stress treatments and provides an estimation of probable cross-tolerances between various sublethal stresses. The obtained data was structured by taking into account differences between groups with a higher consideration, sorting the obtained mass spectra and grouping responses based on their similarity. In general, the separation of stress responses is clearly demonstrated, although the analysis revealed significant differences to any other treatment only for alkaline pH stress. Interestingly, stress responses of F19 to osmotic salt stress (NaCl, KCl) differentiated from osmotic sugar stress (lac, suc). Along with these findings, F19 reacted to temperature stress with the same stress response, regardless whether heat or cold stress was applied. The pool of accumulated data concerning stress responses and adaptions is numerous and immense, as even on species level the stress responses are diverse [73]. Nevertheless, the differentiation of microbial responses in LAB induced by sublethal stresses is logical, since it is currently known that environmental stress responses depend on the stress type applied. Environmental stresses evoke adaptive responses which also induce (cross) tolerances to other adaptive responses than the applied one and additionally raise the bacterial stress tolerance [7].

The detected overlap of sublethal heat, cold, acid and osmotic sugar stress in this study is plausible, since adaptive responses to acid stress can evoke cross tolerances to other stresses such as osmotic and heat stress [74–77]. Moreover, cold stress probably acts as enhancer for thermo-tolerance and thus reveals similarities in regulatory mechanisms between cold- and heat-stress [18, 78, 79]. Moreover it is known, that induced glucose-starvation increases the tolerance to various stress conditions, such as heat, osmotic and acid stress [19, 80]. Furthermore, in *Escherichia coli* and *Bacillus subtilis* general stress proteins controlled by the sigma factor σ^B^ are induced in numerous stress conditions, particularly in glucose-starvation conditions [81, 82]. Nevertheless, in LAB, σ^B^ has so far not been found, but this study enables a first attempt to characterize the stress response to glucose-starvation.

The grouping of sublethal oxidative and osmotic (NaCl, KCl) stress is explainable. Based on studies in *Enterococcus faecalis* JH2-2 and *Lactococcus lactis* subsp. *lactis* IL1403, the microbial response to oxidative stress has so far been connected to heat or acid stress [19, 80]. Nonetheless, Ward *et al*. demonstrated a specific osmotic stress response in *Lactobacillus sanfranciscensis* based on the accumulation of 3-methylbutanoic acid [83]. Under osmotic stress, *Lb*. *sanfranciscensis* synthesizes 3-methylbutanoic acid which is NAD+ dependent, helping to increase the internal pH and to maintain the NAD+/NADH balance. The accumulation of 3-methylbutanoic acid was additionally observed under oxidative stress [84]. In addition, constant, intracellular homeostasis in forms of ion composition, pH and metabolite levels are of great importance for bacteria in order to perform active metabolism [85]. So, for instance, under osmotic stress, bacteria accumulate compatible solutes or stabilizing enzymes keeping the intracellular homeostasis, which additionally protect the cells even against drying [86].

Unfortunately, the identification of biomarkers related to fitness and stress proteins with MALDI-TOF MS itself is limited. Characteristic protein expression profiles are recorded in a lmw range of 2 kDa to 20 kDa and are usually attributed to peaks derived from ribosomal and cell structure proteins [63]. However, to confirm the usage of MADLI-TOF MS for the comprehensive screening and analysis of stress responses, we compared the proteome of F19 cultured in reference conditions and osmotic potassium chloride stress conditions (1M KCl) by 2D GE coupled to ESI MS/MS. In general, overall 20 protein spots were over- and repressed in a defined, sublethal stress. Consequently, variations of protein expression levels as a result of stress induction are connected to the organism’s stress response, which has been shown in several studies [75, 87–90].

Spot PO2 showed homology to CTP-synthase, which is part of the pyrimidine pathway. CTP-synthase, derived from the pyrG gene, catalyses the ATP-dependent transfer of the amide nitrogen from glutamine to the C-4 position of uridine triphosphate (UTP) to generate cytidine triphosphate (CTP) [91]. Accordingly, CTP synthase was shown to hold a substantial role in the pyrimidine metabolism for the biosynthesis of ribo- and deoxiribonucleotides [92]. Since the induction of stress can cause RNA and DNA degradation, the upregulation of CTP-synthase in the presence of high external salt concentration is understandable in order to perform nucleotide rescue necessary for DNA repair. Furthermore, Carman and Henry reported that in the yeast *Saccharomyces cerevisiae*, CTP-synthase plays a major role as precursor of the CDP-base intermediates, which are essential for the biosynthesis of membrane phospholipids [93]. In addition, Ingerson-Mahar reported lately that in *Caulobacter crescentus* CTP-synthase forms cytoskeletal filaments [94]. Considering both findings, CTP-synthase probably plays an additional role in maintaining cellular shape besides its function in the pyrimidine metabolism.

The enzyme alanine-phosphoribitol ligase was also accumulated after osmotic potassium chloride stress. Alanine-phosphoribitol ligase uses the substrates ATP, D-alanine and poly (ribitol phosphate) and converts them to the products AMP, diphosphate and O-D-alanyl-poly (ribitol phosphate) and is thus involved in the synthesis of poly ribitol teichoic acids [95]. This is logical, since teichoic acids are found in the cell wall of gram-positive bacteria and it is known, that in absence of teichoic acids bacteria are more sensitive to an environment with high salt content. Concluding teichoic acids are important for the osmotic stress tolerance [96–100].

Spot P12 was identified as one of the two single-stranded DNA-binding proteins (SSB) coded in the genome of F19. SSB proteins are globally found in living organisms and interact with ssDNA in order to prevent both from forming secondary structures, from premature annealing and from the ssDNA from being digested by nucleases. Thus, SSB are important in DNA replication, recombination and repair, which has been shown in several studies [101, 102]. Under imposed stress conditions, it is obvious that DNA repair and protection responses are needed for the survival of the cell. To our knowledge, so far the upregulation of SSB proteins was described as response to an induction of acid stress [103–105]. However, the accumulation of the SSB protein as response to an increase of external osmolality indicates its importance for the osmotic stress tolerance.

In hypertonic environments, microorganisms require specific strategies to maintain cell turgor (osmoadaptation) in order to survive and perform growth in these conditions. Bacteria cope the changes in external osmolality by accumulating organic osmolytes such as glycine-betaine, glutamate, alanine and proline [106–108]. Accordingly, the upregulation of proline iminopeptidase (PepI) after an increase in extracellular KCl is understandable. Belonging to the family of peptidases, PepI catalyses the hydrolysis of N-terminal amino acid, preferably proline, from a peptide [100, 101]. The release of proline into the cell followed by the accumulation indicates an increase in osmotic stress tolerance. Furthermore, besides the synthesis of osmolytes, Prasad reported that *Lb*. *plantarum* relies on the uptake of compatible solutes from the culture medium, which is done by ABC transporters [90]. These results are in accordance to our findings of an upregulated glutamine ABC transporter ATP-binding protein. The ABC transporter hydrolyses ATP to actively transport substrates such as ions, sugars, lipids, peptides, proteins, implying osmolytes accumulation.

The identification of probable stress proteins, which were obtained by 2D GE coupled to ESI MS/MS, confirm the approach of using MALDI-TOF MS for the fast and comprehensive analysis of stress responses. To our knowledge, this is the first report of the implementation of MALDI-TOF MS protein profiling for a substantial analysis of various stress responses in LAB. Thus, we generally consider MALDI-TOF MS analysis as an excellent analytical tool for an easy identification of maximal stress responses and proximately exploit these conditions since they induce tolerances against stress. In the manufacture of foods, in which starter cultures are subjected to various unfavourable environmental stress conditions, a maximal survival and fitness of starter cultures induced by stress tolerances is desired. Therefore, the process of preconditioning is used. In this context, MALDI-TOF MS analysis can be used to find optimal conditions for the preparation of starter cultures.

## Supporting Information

S1 Fig MALDI-TOF mass spectra obtained for *Lb*. *paracasei* subsp. *paracasei* F19 in the mass range from 2 000 Da to 15 000 Da. Control conditions (control) and sublethal stress conditions at different sampling points are indicated; dotted lines mark peaks with increasing or decreasing peak intensity.
(TIF)

S2 Fig Cluster analysis of *Lb*. *paracasei* subsp. *paracasei* F19 after induced sublethal stress conditions. X-axis indicating different sampling points; y-axis displays distances indicating similarity of spectra from 0 (identical spectra) to 1 (maximum variability); A, displays analogies among cluster formation of NaCl, pH9 and lac stress; B, displays analogies among cluster formation of KCl, glu10, pH4, suc, 15°C, 45°C and H2O2 stress.
(TIF)

S1 Table Probable proteins identified from 2D gel electrophoresis. Displayed are the analysed protein spots from 2D gel electrophoresis (sample), the probable protein identifications (Protein name) including the protein identification probability (%), sequence coverage (%), peptide sequence(s), exclusive unique peptide and spectrum count and total spectrum count in accordance to the accession and KO no.
(DOCX)
