# Supplementary figures and images for: MALDI-TOF Mass Spectrometry Enables a Comprehensive and Fast Analysis of Dynamics and Qualities of Stress Responses of Lactobacillus paracasei subsp. paracasei F19
Source: PLoS One. 2016 Oct 26;11(10):e0165504. doi: 10.1371/journal.pone.0165504 (PMC5082675; doi:10.1371/journal.pone.0165504)

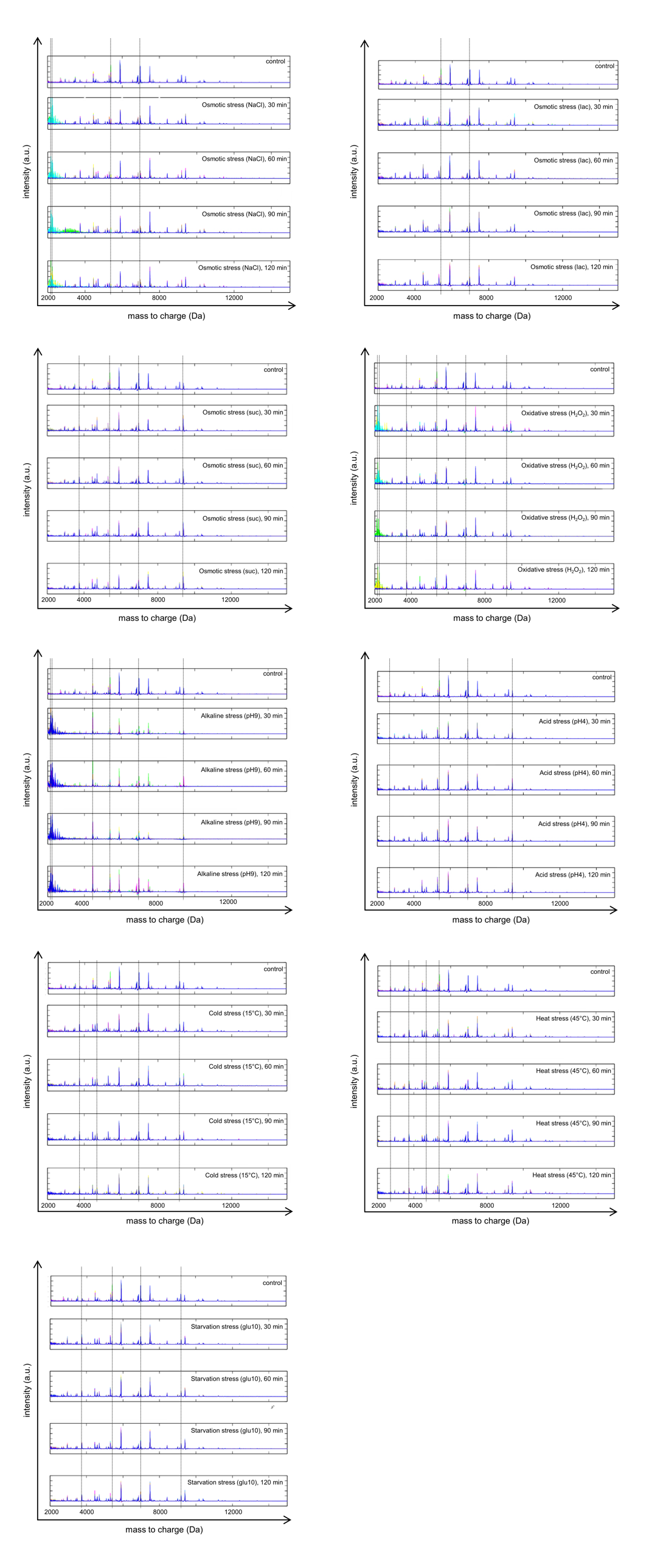

Supplement: S1 Fig — Control conditions (control) and sublethal stress conditions at different sampling points are indicated; dotted lines mark peaks with increasing or decreasing peak intensity. (TIF) [file pone.0165504.s001.tif]
