# Supplementary material for: MALDI-TOF Mass Spectrometry Enables a Comprehensive and Fast Analysis of Dynamics and Qualities of Stress Responses of Lactobacillus paracasei subsp. paracasei F19
Source: PLoS One. 2016 Oct 26;11(10):e0165504. doi: 10.1371/journal.pone.0165504 (PMC5082675; doi:10.1371/journal.pone.0165504)
Supplement: S1 Table — Displayed are the analysed protein spots from 2D gel electrophoresis (sample), the probable protein identifications (Protein name) including the protein identification probability (%), sequence coverage (%), peptide sequence(s), exclusive unique peptide and spectrum count and total spectrum count in accordance to the accession and KO no. (DOCX) [file pone.0165504.s003.docx]

S1 Table: Probable proteins identified from 2D gel electrophoresis. Displayed are the analysed protein spots from 2D gel electrophoresis (sample), the probable protein identifications (Protein name) including the protein identification probability (%), sequence coverage (%), peptide sequence(s), exclusive unique peptide and spectrum count and total spectrum count in accordance to the accession and KO no.

| Sample | Protein name | Accession no. | KO no. | Protein identification probability (%) | Sequence coverage (%) | Peptide sequence(s) | Exclusive unique peptide count | Exclusive unique spectrum count | Total spectrum count |
| --- | --- | --- | --- | --- | --- | --- | --- | --- | --- |
| PO2 | CTP synthase | BBD24_12645 | K01937 | 100.0 | 12.7 | AFIQAAGDFKA  AYDNAHEIQKR  DQVGVKNLGGTLR  GLEGKIAAIR  LMEIIEYPK  LMEIIEYPKNK  SIGIQPNMLVVR | 6 | 8 | 9 |
| PO8 | proline iminopeptidase | BBD24_12800 | K01259 | 100.0 | 24.7 | QLIMYDQVGCGK  SSLPEDPAVYVK  SVMIDGSPSSIK  TVVDHIPNAK  YLAANDRYMEK  YLSYEDREAIAEAER | 5 | 5 | 6 |
| PO9 | alanine-phosphoribitol ligase | BBD24_05660 | negative | 100.0 | 11.7 | FAVTLITGK  LANPMEAEAAYFAK  MLKLGVIGTGWITK | 2 | 2 | 3 |
| PO11 | glutamine ABC transporter ATP-binding protein | BBD24_07020 | K02028 | 99.6 | 4.4 | TQDFLSKILAQ | 1 | 1 | 1 |
| PO12 | single-stranded DNA-binding protein | BBD24_00055 | K03111 | 100.0 | 17.2 | GSMVGVEGHIQTR  KGSMVGVEGHIQTR  MLNSVALTGR  SAENFANFTKK | 4 | 4 | 4 |
